# The Impact of Depression and Malnutrition on Health-Related Quality of Life Among the Elderly Iranians

**DOI:** 10.5539/gjhs.v7n3p161

**Published:** 2014-11-25

**Authors:** Sareh Keshavarzi, Seyed Mehdi Ahmadi, Kamran B. Lankarani

**Affiliations:** 1 Department of Epidemiology, Faculty of Health, Shiraz University of Medical Sciences, Shiraz, Iran; 2 Health Policy Research Center, School of Medicine, Shiraz University of Medical Sciences, Shiraz, Iran

**Keywords:** depression, elders, health-related quality of life, Iran, malnutrition

## Abstract

**Introduction:**

The present study aimed to assess the association between nutritional status and depressive symptoms among elderly Iranians and to explore their impact on their Health-Related Quality of Life (HRQoL).

**Methods:**

In this cross-sectional study, 447 elders aging from 55 to 85 years were randomly selected and completed the Iranian version of Geriatric Depression Scale-15 (GDS), Mini Nutritional Assessment (MNA), and the Iranian version of Short Form Health Survey (SF-36).

**Results:**

Out of the 447 elderly, 72.1% were female with the mean age of 65.99 ± 7.89 years. The prevalence of depression was 38.1%. In addition, the SF-36 sub-scores tended to be lower among the elders with depressive symptoms according to GDS. The Physical Functioning (PF), Bodily Pain (BP), Role Physical (RP), Role Emotional (RE), and Mental Health (MH) dimensions of the SF-36 were also statistically poorer in the elders with depression. The mean MNA score was 24.6 ± 2.7; 35.4% of the participants were malnourished or at risk of malnutrition and 64.6% were adequately nourished. The sub scores of SF-36 were significantly lower in the elders with impaired nutritional status.

**Conclusions:**

Considering the importance of the association among psychological and nutritional problems and HRQoL in caring for and promoting the welfare of the elders, this study provided fundamental information and a basis for further evaluation of this issue in developing and undeveloped countries.

## 1. Introduction

Aging is an unavoidable process of every living organism and is related to a reduction in the homeostatic control and reserve capacity of the organ systems, the capability to adapt to the environmental factors, and the capacity of a stress response. In addition to being an unavoidable physiologic process, aging is one of the most important causes of decrease in life quality due to its chronologic, biological, social, and psychological dimensions (DeLisa et al., 2005; Power et al., 1998). Assessment of quality of life has played an important role in development of health services (Keshavarzi et al., 2012; Klassen et al., 1996). Depression is the most frequent health problem in aged individuals that may significantly affect their quality of life (Blazer, 2003). Epidemiological studies have indicated the prevalence rates of depression in free living elderly to range from 10% to 15%, and it can reach up to 45% in institutionalized individuals (Jongenelis et al., 2004; Webber et al., 2005; Teresi et al., 2001). It is commonly observed that depressed elderly patients are more likely to have worse general health along with a significant decrease in their quality of life (Shmuely et al., 2001).

The other common problem in late life is malnutrition with the prevalence rate of up to 60% in the institutionalized elders. Malnutrition has serious unfavorable effects on health and one of its correctable causes is depression (Vellas et al., 2001; Saletti et al., 2000; Suominen et al., 2005). In other words, a reduced energy intake causing body weight loss may be caused by social or physiological factors or a combination of both. Depression in the elderly can often lead to malnutrition or dehydration which in turn can induce various kinds of physical illnesses. On the other hand, physical conditions, such as malnutrition, in the elderly can induce depression because of their psychological vulnerability. Due to the strong relationship between the body and the mind in the elderly, psychiatric care seems to be necessary (Wada, 2000). One of the most common causes of depression which leads to psychological issues and malnutrition in the elderly is related to the experience of loss or breakage of social networks (Pirlich & Lochs, 2001; Chapman, 2006; Cederholm & Hellström, 1995; Donini et al., 2003). Several studies have indicated that depression was a major factor contributing to weight loss in the elderly and malnourished patients had higher depression scores (Morley & Kraenzle 1994; Blaum et al., 1995). However, the casual relationship between depression and nutritional status is still unclear (Singh et al., 2014).

A higher prevalence of untreated depression, malnutrition, and disabilities in the elderly compared to other age groups and the subsequent limitations in their social activities negatively affect the quality of life and well-being and, consequently, can increase mortality. Currently, there is a growing number of research and increased clinical interest in developing robust quantitative measures of quality of life that can be used in clinical assessments and economic models. Moreover, the increase in the elderly population makes it necessary to investigate the characteristics of depression whose presence adversely affects the social interaction and may cause malnutrition, decrease the quality of life, cause pain and discomfort, and have a significant economic impact due to the significantly high treatment costs (Bergquist & Frantz, 2001).

In public health and medicine, the concept of quality of life refers to an individual’s perception of happiness and satisfaction with life and position in life in the context of the culture and value systems in which they live and in relation to their expectations, values, and concerns (Power et al., 1998). The impacts of depression and malnutrition on the quality of life should be assessed and monitored. Instruments that assess Health-Related Quality of Life (HRQoL) measure the degree to which functional, physical, mental, and social aspects are impaired by symptoms, incapacities, and limitations caused by diseases (Hazzard et al., 2003). HRQoL can be measured by either generic or specific instruments that, for the most part, were originally developed in English language, translated into other languages, and validated for different cultures (McDowell et al., 2006; Ware Jr & Gandek, 1998). The SF-36 questionnaire (Medical Outcomes Trust, Waltham, Massachusetts, United States) was translated and validated in Iran by Montazeri et al., (2005) in a population-based study on 4163 healthy individuals aging 15 years and above (Montazeri et al., 2005). It was considered as an appropriate instrument with regard to the socioeconomic and cultural characteristics of the study population. This instrument measures several dimensions of health and assesses the impact of diseases and the benefits of treatment. It is a generic HRQoL instrument composed of 36 items organized into eight health concepts, including physical function, social and psychological factors, life satisfaction, well-being, and awareness of health status (McDowell et al., 2006; Ware Jr & Gandek, 1998).

The impact of nutrition on health and functional status of the elderly is well-known and studies in hospitalized and community dwelling elderly have shown that depression increased the risk of impaired nutritional status. However, there exists little information on these disorders among the elders in the community and their impact on the quality of life of this population especially in developing countries. Moreover, health maintenance and improvement in quality of life in the elderly population can be possible by implementation of recommendations based on scientific research. Thus, the present study aims to assess the nutritional status and the prevalence of depression and examine the association between these health-related problems and quality of life in the elders using SF-36. Furthermore, the effects of socio-demographic, medical, and emotional factors on the participants’ quality of life were evaluated through a cross-sectional assessment. Our hypothesis was that the presence of depressive symptoms and malnutrition would have a significant negative impact on HRQoL.

## 2. Materials and Methods

A total of 447 non-institutionalized individuals above 55 years of age were recruited into the present study. In general, old age is defined according to a range of characteristics, including chronological age, change in social role, and changes in functional abilities. In high-resourced countries, old age is generally defined in relation to retirement from paid employment and receipt of a pension at 60 or 65 years of age. With increase in longevity, some countries have defined a separate group of the oldest people; i.e., those over 85 years old. In low-resourced situations with shorter life-spans, old people may be defined as those over 50 years of age. The age of 50 years was accepted as the definition of old people for the purpose of WHO Older Adult Health and Ageing in Africa project (WHO, 2010).

In the present study, the subjects were selected using two-stage cluster sampling. All the subjects signed written informed consents and the study was approved by the local Ethics Committee.

The study data were collected using Mini Nutritional Assessment (MNA), the Iranian version of Geriatric Depression Scale-15 (GDS), and the Iranian version of Short Form Health Survey (SF-36).

MNA is a valid instrument which can be used for identifying the geriatric patients who are malnourished or at risk of malnutrition (Barone et al., 2003). It is both a screening and assessment instrument for determination of malnutrition in the elderly. This instrument eliminates the need for more invasive tests, such as blood sampling. It consists of items regarding general condition, appetite, anthropometry, food preference, and subjective perception of health status. In case a patient gains 12 points or more in the screening part of MNA, s/he is not at risk and is not required to complete the rest of the questionnaire (for collecting more data in the present study, the questionnaire was filled out completely). On the other hand, if a patient obtains 11 points or less, s/he may be at risk and the whole MNA should be completed. Based on the final scores, the study patients were categorized into three groups: scores below 17 (malnutrition), scores between 17 and 23.9 (at risk of malnutrition), and scores between 24 and 30 (well nourished).

At the study center, the participants who were instructed to wear no shoes and light clothing had their weight (kg) and height (cm) measured to the nearest decimal using a Jenix DS-102 stadiometer (Dong Sahn Jenix Co., Ltd., Seoul, Korea). In addition, Body Mass Index (BMI) was calculated as weight divided by height squared (kg/m2).

GDS is a screening tool to identify the elderly patients who are at risk of depression. This screening instrument includes 30 yes/no items evaluating the level of depressive symptoms within the last week. This self-estimated scale was developed by Yesa-vage et al., (Yesavage et al., 1983) and the validity and reliability of its Persian version have been confirmed (Malakouti et al., 2006). In this scale, high scores indicate a high level of depressive symptoms. According to GDS, in case a patient gains 8 points or less, s/he is not at risk (Yesavage et al., 1983).

In this study, HRQoL was evaluated using the Iranian version of SF-36 questionnaire (Montazeri et al., 2005). This questionnaire consists of 8 subscales namely Physical Functioning (PF), Role Physical (RP), Bodily Pain (BP), General Health (GH), Vitality (VT), Social Functioning (SF), Role Emotional (RE), and Mental Health (MH). PF, RP, and BP subscales measure physical limitations in daily life resulting from decreased functional status and pain. In addition, VT and GH represent the personal perception of the global health status. Finally, SF, RE, and MH evaluate anxiety as well as the effect of emotional problems on daily life and social contacts. The scores of this instrument range from 0 to 100 for each subscale, with higher scores representing a more desirable state. These scales range from 0-100 percent (absolute values), while the norm values differ substantially across the scales. Thus, to facilitate the interpretation and to compare the results with the norm values obtained in 1998 (1998 SF-36 US population norms), all the scales were not only expressed as absolute values, but also as norm-based scores. This means that each scale was scored to have the same mean and standard deviation. In the current study, the standard (US) scoring algorithms were used as recommended by Ware et al., (1998). Hence, the results of the eight scales can directly be compared because they are all standardized relative to the population norms.

In this study, all the questionnaires were administered through face to face interviews. The interviewers were trained and calibrated. Admission and data analysis was followed by examining the relationship between malnutrition and depression as well as education, gender, working status (employed, unemployed, and retired), BMI, and doing exercise. The effects of MNA and GDS scores and also some socio-demographic variables (such as education, gender, and working status) on the elders’ quality of life were investigated, as well.

All the statistical analyses were carried out using the SPSS statistical software (version19; SPSS Inc., Chicago, IL, USA). Normal distribution of the variables was assessed using Kolmogorov-Smirnov test. The results of descriptive statistics were expressed as means ± SDs for continuous variables and as frequencies and percentages for categorical variables. The subjects with and without depression or malnutrition were compared by Chi-square test for categorical variables, including gender and years of education, and independent sample t-tests for continuous variables, such as different subscales of HRQoL. Moreover, Pearson correlation coefficients were calculated for the linear relations between the subscales of HRQoL and continuous variables, such as MNA scores, GDS score, and the measures of BMI. In order to identify the factors affecting the subjects’ quality of life, a two-step regression analysis with adjustment of the possible confounding effects was conducted for each SF-36 subscale. Univariate analyses were initially performed and the variables that reached a value of P<0.15 were considered as the potential factors and incorporated into the second phase of the multivariate analysis to identify the factors associated with SF-36 scores. Besides, a two-sided P-value<0.05 was considered as statistically significant.

## 3. Results

The mean (SD) age of the study population (n=447) was 65.99 (7.89) years. In addition, 322 subjects (72.1%) were female, 380 (85%) had ≤12 years of education, and only 55 (12.2%) were employed. The elders’ mean score of depressive symptoms as defined by GDS score was 9.04 (6.96). Besides, the prevalence of depression as defined by GDS score >8 was 38.1%. Table 1 presents the demographic and nutritional parameters of the study subjects based on the evaluation by GDS. The findings of the study revealed a significant difference between the depressed and non-depressed elderly regarding age, gender, years of education, working status, regular exercising, and MNA score. Moreover, the prevalence of depression was higher in females compared to males. Also, the subjects with depressive symptoms had a significantly worse nutritional status, lower level of education, and lower ages.

**Table 1:** The subjects’ characteristics according to evaluation by Geriatric Depression Scale (GDS)

Characteristics	Overall study population (n=447)	Depressed (n=170)	Non-depressed (n=277)	P-value
**Gender (%)**				
**Male**	125(27.9)	30(17.9)	95(34.1)	0.038
**Female**	322(72.1)	140(82.1)	182(65.9)	
**Years of Education (%)**				
**>12 years**	67(15)	3(1.8)	64(23.1)	<0.001
**Years of Education (%) >12 years ≤12 years**	380(85)	167(98.2)	213(76.9)	
**Working Status (%)**				
**Employed**	55(12.2)	9(5.4)	46(16.5)	<0.001
**Unemployed**	222(49.7)	121(71.4)	100(36.3)	
**Retired**	170(38.1)	40(23.2)	131(47.3)	
**Regular Exercise (%)**				
**Yes**	295(66)	91(53.6)	204(73.6)	0.013
**No**	152(34)	79(46.4)	73(26.4)	
**Age (Mean ± SD)**	65.99±7.89	63.95±7.32	67.24±8.00	0.013
**BMI (kg/m^2^)(Mean ± SD)**	28.27±5.31	29.01±4.60	27.81±5.68	0.182
**MNA (Mean ± SD)**	24.56±2.97	22.56±2.98	25.78±2.20	<0.001

Table 2 shows the demographic and nutritional parameters of the study population according to the nutritional status. The mean (SD) score of MNA was 24.56 (2.97). Besides, 158 elders (35.4 %) were malnourished or at risk of malnutrition, while 289 (64.6%) were well-nourished. As anticipated, however, the malnourished elders exhibited higher GDS scores compared to the well-nourished ones. Nonetheless, no significant difference was observed between well and malnourished elders concerning age, gender, and BMI.

**Table 2:** The subjects’ characteristics according to the nutritional status

Characteristics	Malnourished or at risk (n=158)	Well-nourished (n=289)	P-value
**Gender (%)**			
**Male**	33(21.2)	91(31.6)	0.248
**Female**	125(78.8)	198(68.4)	
**Years of Education (%)**			
**>12 years**	9(5.8)	58(20)	0.028
**≤12 years**	149(94.2)	231(80)	
**Working Status (%)**			
**Employed**	18(11.5)	36(12.6)	0.012
**Unemployed**	103(65.4)	119(41.1)	
**Retired**	37(23.1)	134(46.3)	
**Regular Exercise (%)**			
**Yes**	79(50.0)	216(74.7)	0.002
**No**	79(50.0)	73(25.3)	
**Age (Mean ± SD)**	66.33±7.88	65.80±7.93	0.700
**BMI (kg/m^2^)(Mean ± SD)**	29.28±7.39	27.71±3.64	0.087
**GDS (Mean ± SD)**	13.31±7.71	6.70±5.24	<0.001

Comparison of the mean scores of the 8 domains of SF-36 in the elders with and without depressive symptoms showed that the depressed elders’ HRQoL was significantly lower in RP, PF, BP, RE, and MH domains. Considering other dimensions, however, no significant difference was observed between the depressed and normal elders regarding their SF-36 scores (Table 3). We also compared the mean scores of SF-36 in the elders with and without malnutrition. According to the results, the elders who were malnourished or at risk of malnutrition had significantly worse HRQoL compared to the well-nourished elders in all the SF-36 domains, except for SF (Table 4).

**Table 3:** The subjects’ mean scores of HRQoL measured by the SF-36 according to evaluation by Geriatric Depression Scale (GDS)

SF-36 Scales	Overall study population (n=447)	Depressed (n=170)	Non-depressed (n=277)	P-value
	**Mean ± SD**	**Mean ± SD**	**Mean ± SD**	
**Physical subscales**				
**Physical functioning (PF)**	23.82±5.52	22.11±5.82	24.88± 5.08	0.004
**Role physical (RP)**	17.16±3.68	16.05±4.43	17.85±2.96	0.009
**Bodily pain (BP)**	3.56±1.90	3.12±1.55	4.27±2.19	<0.001
**Vitality (VT)**	12.09±2.22	11.80±2.53	12.26±2.01	0.251
**Mental subscales**				
**General health (GH)**	14.46±2.65	14.28±14.73	14.73±2.54	0.323
**Social functioning (SF)**	5.88±0.85	5.82±1.19	5.91±0.55	0.532
**Role emotional (RE)**	12.51±2.99	11.25±3.54	13.28±2.29	<0.001
**Mental health (MH)**	15.90±2.36	15.21±2.83	16.33±1.91	0.011

**Table 4:** The elders’ mean scores of HRQoL measured by SF-36 according to the nutritional status

SF-36 Scales	Malnourished or at risk (n=158)	Well-nourished (n=289)	P-value

	Mean ± SD	Mean ± SD	
**Physical subscales**			
**Physical functioning (PF)**	21.62±5.78	25.03±5.01	0.001
**Role physical (RP)**	15.48±4.56	18.08±2.72	<0.001
**Bodily pain (BP)**	3.22±1.70	4.17±2.09	0.006
**Vitality (VT)**	11.54±2.42	12.39±2.06	0.026
**Mental subscales**			
**General health (GH)**	14.10±2.57	15.10±2.70	0.03
**Social functioning (SF)**	5.87±0.99	5.88±0.77	0.809
**Role emotional (RE)**	11.81±3.17	12.89±2.83	0.035
**Mental health (MH)**	15.37±2.55	16.26±2.05	0.032

Linear regression analyses identifying the factors associated with different subscales of HRQoL have been presented in Table 5. In summary, the results showed that after adjustment for other variables, the effects of depressive symptoms persisted for 4 domains of SF-36; i.e., PF, BP, RE, and MH (*P*<0.05). Another important factor that had a negative effect on HRQoL was “nutritional status”. In comparison to the well-nourished elders, those who were malnourished or at risk of malnutrition had significantly worse HRQoL in the following domains: PF (*P*<0.05), RP (*P*<0.05), and VT (*P*<0.05). In addition, being unemployed had a significantly negative effect on VT (*P*<0.01) and higher age had a significantly negative effect on PF (*P*<0.001). Furthermore, male gender had a negative effect on PF (*P*<0.05) but a positive effect on VT (*P*<0.05). Another important factor that had a positive effect on HRQoL was “regular exercising”. In comparison to the elders who never exercised, those who exercised regularly had significantly better PF (*P*<0.05). However, with regards to the years of education and BMI, no significant effect was seen on the HRQoL subscales.

**Table 5:** Linear regression analysis of the SF-36 scores

Independent Variables	Regression Coefficient of the SF-36 Subscales (P-value)¦							

	PF	RP	BP	VT	GH	SF	RE	MH
Gender (reference: female)	-2.280*	-0.397	0.096	0.990*	0.514	NS	-0.353	NS
Years of education (reference: >12 years)	NS	NS	NS	NS	-0.359	NS	NS	NS
Working status (reference: employed)	NS					NS		NS
**Unemployed**		0.738	-0.376	-1.257*	-0.398		0.284	
**Retired**		-0.627	0.290	0.742	0.318		-0.421	
Regular exercise (reference: yes)	-1.930*	-0.873	NS	NS	NS	NS	NS	-0.363
Age	-0.221‡	NS	NS	NS	NS	NS	NS	NS
BMI	-0.043	NS	0.025	-0.05	0.049	NS	NS	NS
With depressive symptoms	-2.008*	-0.396	-0.730*	NS	NS	NS	-1.694†	-0.892*
With malnutrition/at risk of malnutrition	-1.670*	-1.855†	-0.437	-0.764*	-0.640	NS	-0.166	-0.368
R-square (Adjusted)	0.216	0.133	0.104	0.077	0.051	-	0.100	0.045

* *P* <0.05,

† *P* <0.01,

‡ *P* <0.001.

¦ Adjusted for all other variables in the table except for those marked with “NS”. NS – Not significant in univariate analysis thus not included in the linear regression for the quality of life scores. PF: Physical functioning; RP: Role limitations due to physical problems; BP: Bodily pain; GH: General health; VT: Vitality; SF: Social functioning; RE: Role limitations due to emotional problems; MH: Mental health.

## 4. Discussion

HRQoL has received increasing interest in epidemiology and health outcomes research recently. In the present study, we investigated the quality of life of 447 elders in relation to their evaluated GDS scores and their nutritional status. Our findings showed that both malnutrition and depressive symptoms were highly prevalent among the geriatric population. The large number of the individuals identified as malnourished or at nutritional risk is in concordance with the literature as is the number of the participants with depressive symptoms (Kvamme et al., 2011; Ahmadi et al., 2013; Eriksson et al., 2005; Norman et al., 2006; Smoliner et al., 2009). The findings of the current study showed that HRQoL was significantly reduced in the individuals with impaired nutritional status. Moreover, the perception of physical ability and various components of quality of life were impaired in the participants with depressive symptoms. As expected, the elderly individuals who reported depression presented HRQoL that was affected by all the subscales, except for VT, GH, and SF. The HRQoL of the elders suffering from depression was most evident on the RE, BP, and PF subscales. The damaging effect of emotional status was profound, and the fact that emotional condition significantly affected bodily pain was noteworthy, as well. Pihl et al., also found that among the elderly patients suffering from depression, the greatest losses were in the PF, BP, and GH areas (Pihl et al., 2005). On the other hand, Smoliner et al., reported the largest differences in RP and MH (Norman et al., 2006). Thus, providing the depressed elderly patients with adequate care can help reduce suffering as well as its impacts on the quality of life.

In comparison to the well-nourished elders, those who were malnourished or at risk of malnutrition experienced a significantly lower quality of life scores, particularly in PF and RP scales. Of course, our findings regarding the poorer HRQoL among the elders with malnutrition or risk of malnutrition compared to the well-nourished ones were not unexpected. Other studies have also shown these subscales as the most affected ones among the patients with malnutrition (Kvamme et al., 2011; Ahmadi et al., 2013; Eriksson et al., 2005; Norman et al., 2006). Norman et al., found the greatest reductions to be in the RP, MH, and VT subscales (Norman et al., 2006). In Berlin, Smoliner et al., studied 114 residents of a nursing home and found the lowest means of the SF-36 values in GH and VT. On the other hand, Verma et al., reported the greatest reductions in MH and RE (Smoliner et al., 2009; Pihl et al., 2005).

It has been well documented that HRQoL in general population was influenced by socio demographic factors, such as age, gender, education level, physical activity, and marital status. Fewer problems on the descriptive system and higher scores in the subscales of HRQoL were most prevalent among the subjects who were younger, male, had higher education levels, were married, and did exercise (Quercioli et al., 2009; Cherepanov et al., 2010). Not surprisingly, our study findings revealed better PF among the younger patients compared to the older ones. It was also not surprising to find a positive association between regular exercising and the PF domain of HRQoL. However, by far the most significant negative impact on all the subscales of HRQoL was due to the presence of depressive symptoms and malnutrition. Similar findings have been reported in the previous studies, as well (Kvamme et al., 2011; Ahmadi et al., 2013; Eriksson et al., 2005; Norman et al., 2006; Smoliner et al., 2009; Pihl et al., 2005; Verma et al., 2010).

The results of the current study emphasized the need for better organization of quality in healthcare services for the chronic conditions in the elderly. In fact, such improvements can help prevent the complications of these diseases. Hence, healthcare services should become more effective in management of the disorders, such as depression and malnutrition, which lead to various disorders that have a negative effect on autonomy and wellbeing. Moreover, the high prevalence of depression and malnutrition which are inevitable components of the aging process requires improvements and adjustments in prevention, control, and treatment procedures.

Furthermore, along with the adequate medical care for the elderly patients, screening programs should also be established for early diagnosis of the aforementioned conditions. Besides, patients should be monitored regularly after diagnosis for treatment compliance. Of course, health disparities should be offset by paying more attention to the elderly with lower socioeconomic statuses.

The importance of the present study comes from the fact that it is the first study on the Iranian elders quantifying the impact of depression, malnutrition, and a number of socio-demographic conditions on the eight areas of HRQoL assessed by SF-36. The study results were similar to those obtained in other countries, and there is general agreement regarding the mostly affected subscales.

This study had some limitations. One of the limitations of the present study was that no inference could be made regarding the causal relationship among nutritional status, depression, and quality of life because the study followed a cross-sectional design. Yet, the study findings draw our attention to the fact that HRQoL that looks at health from an individual’s perspective is truly multi-faceted, and simple measures to detect and treat depression can go a long way towards reducing the burden of malnutrition and disability. Further studies are needed to identify the direction of this relationship and to assess the effect of depression treatment on nutritional status as well as on HRQoL.

## 5. Conclusion

Overall, the findings of the present study revealed the need for interventions that consider the effect of depression and malnutrition on different dimensions of HRQoL, with particular attention to the elders. Moreover, the effect of such disorders on HRQoL subscales should be evaluated occasionally in order to assess the improvements in healthcare and social services for the elderly individuals.
